# Supplementary material for: Highly Sensitive Detection of PQS Quorum Sensing in Pseudomonas Aeruginosa Using Screen-Printed Electrodes Modified with Nanomaterials
Source: Biosensors (Basel). 2022 Aug 13;12(8):638. doi: 10.3390/bios12080638 (PMC9406015; doi:10.3390/bios12080638)
Supplement: Supplementary file 1 [file biosensors-12-00638-s001.zip › biosensors-1845156-supplementary.pdf]

biosensors

MDPI

Supplementary material

Highly Sensitive Detection of PQS Quorum Sensing in Pseudomonas Aeruginosa using Screen-Printed Electrodes Modified with Nanomaterials

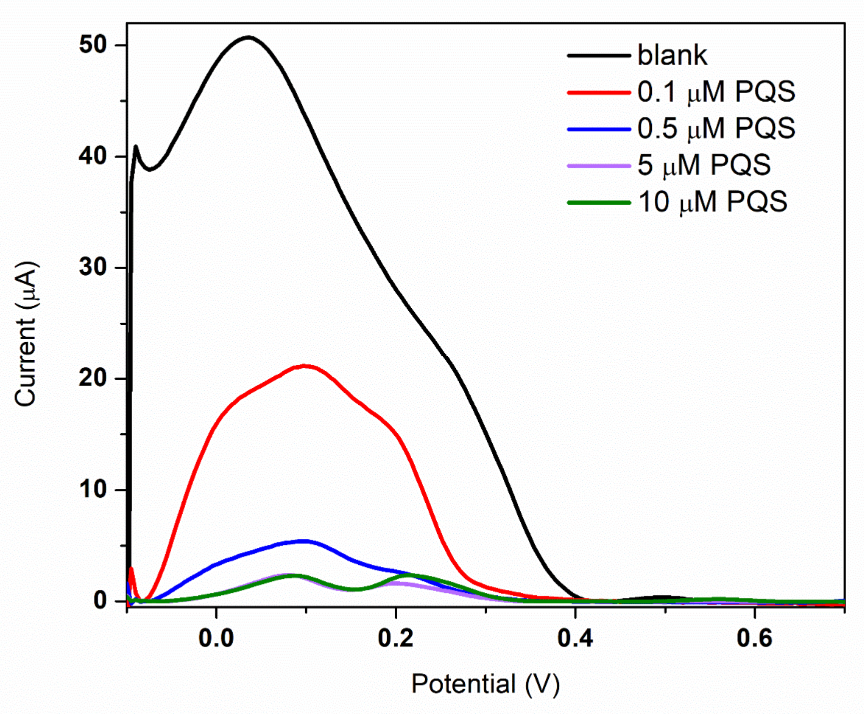

Figure S1. DPV analysis on OMC-SPE for different PQS concentrations in H_2SO_4 0.5 M.

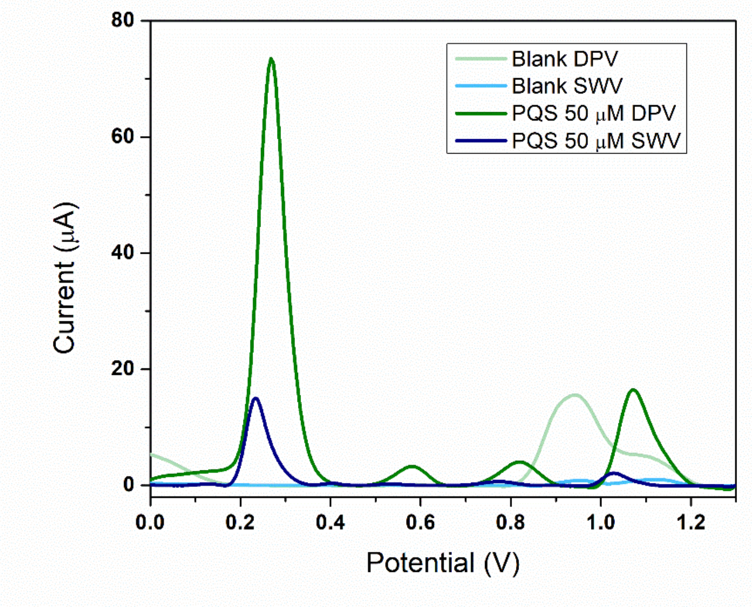

Figure S2. The influence of the electrochemical technique for PQS detection: blank (H_2SO_4 0.5 M) analysis by DPV (light green) and SWV (cyan); analysis of a 50 μM PQS solution by DPV (**dark green**) and SWV (**dark blue**). DPV parameters: SP 0.05 V, MA 0.1 V, MT 0.025 s, IT 0.5 s, SR 0.01 Vs^{-1} . SWV parameters: SP 0.001 V, MA 0.1 V, Frequency 10 Hz, IT 0.5 s, SR 0.01 Vs^{-1}.

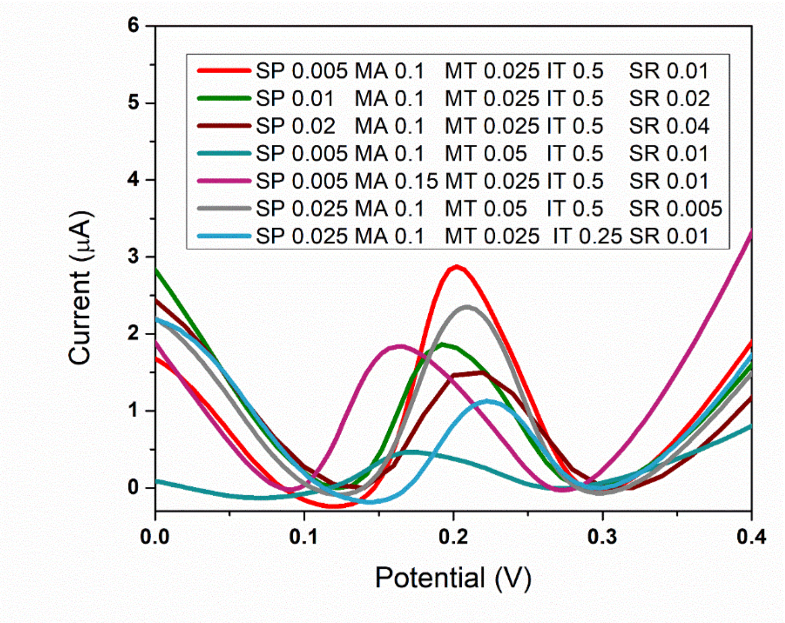

Figure S3. Optimization of the SP (V); MA (V); MT (s); IT (s); SR (Vs^{-1}) in DPV on a −0.1 to 1.3 V PW using a 1 µM PQS solution in H_2SO_4 0.5 M.

Table S1. The optimization of the DPV parameters on a −0.1 to 1.3 V PW using a 1 µM PQS solution in H_2SO_4 0.5 M.

Step potential (V)	Modulation amplitude (V)	Modulation time (s)	Interval time (s)	Scan rate (Vs^{-1})	Current intensity (µA)
**0.005**	**0.1**	**0.025**	**0.5**	**0.01**	**3.02**
0.01	0.1	0.025	0.5	0.02	1.86
0.02	0.1	0.025	0.5	0.04	1.47
0.005	0.1	0.05	0.5	0.01	0.5
0.005	0.15	0.025	0.5	0.01	0.1
0.0025	0.1	0.025	0.5	0.005	2.42
0.0025	0.1	0.025	0.25	0.01	1.22
